# Microbial Community and Functional Structure Significantly Varied among Distinct Types of Paddy Soils But Responded Differently along Gradients of Soil Depth Layers

**DOI:** 10.3389/fmicb.2017.00945

**Published:** 2017-05-29

**Authors:** Ren Bai, Jun-Tao Wang, Ye Deng, Ji-Zheng He, Kai Feng, Li-Mei Zhang

**Affiliations:** ^1^State Key Laboratory of Urban and Regional Ecology, Research Centre for Eco-environmental Sciences, Chinese Academy of SciencesBeijing, China; ^2^Key Laboratory for Environmental Biotechnology, Research Center for Eco-Environmental Sciences, Chinese Academy of SciencesBeijing, China; ^3^College of Resources and Environment, University of Chinese Academy of SciencesBeijing, China; ^4^Faculty of Veterinary and Agricultural Sciences, The University of Melbourne, MelbourneVIC, Australia

**Keywords:** paddy soil, GeoChip, Mi-Seq sequencing, microbial community, soil profile, soil type, network analysis

## Abstract

Paddy rice fields occupy broad agricultural area in China and cover diverse soil types. Microbial community in paddy soils is of great interest since many microorganisms are involved in soil functional processes. In the present study, Illumina Mi-Seq sequencing and functional gene array (GeoChip 4.2) techniques were combined to investigate soil microbial communities and functional gene patterns across the three soil types including an Inceptisol (Binhai), an Oxisol (Leizhou), and an Ultisol (Taoyuan) along four profile depths (up to 70 cm in depth) in mesocosm incubation columns. Detrended correspondence analysis revealed that distinctly differentiation in microbial community existed among soil types and profile depths, while the manifest variance in functional structure was only observed among soil types and two rice growth stages, but not across profile depths. Along the profile depth within each soil type, *Acidobacteria*, *Chloroflexi*, and *Firmicutes* increased whereas *Cyanobacteria*, β*-proteobacteria*, and *Verrucomicrobia* declined, suggesting their specific ecophysiological properties. Compared to bacterial community, the archaeal community showed a more contrasting pattern with the predominant groups within phyla *Euryarchaeota*, *Thaumarchaeota*, and *Crenarchaeota* largely varying among soil types and depths. Phylogenetic molecular ecological network (pMEN) analysis further indicated that the pattern of bacterial and archaeal communities interactions changed with soil depth and the highest modularity of microbial community occurred in top soils, implying a relatively higher system resistance to environmental change compared to communities in deeper soil layers. Meanwhile, microbial communities had higher connectivity in deeper soils in comparison with upper soils, suggesting less microbial interaction in surface soils. Structure equation models were developed and the models indicated that pH was the most representative characteristics of soil type and identified as the key driver in shaping both bacterial and archaeal community structure, but did not directly affect microbial functional structure. The distinctive pattern of microbial taxonomic and functional composition along soil profiles implied functional redundancy within these paddy soils.

## Introduction

Soils cover most of the natural and artificial habitats of terrestrial ecosystems. Due to the high spatial heterogeneity in soil particles and large variation of soil physiochemical properties among soil types, soils are considered harboring the most diverse microbial groups in comparison with other ecosystems (Schimel and Schaeffer, 2012). For a long time, soil scientists have noticed that the soil natural properties determined by soil parent materials during soil formation period, such as pH, texture, and base saturation, etc., sustain soil biodiversity in nature and greatly affect the basic fertility and productivity of soil to a large degree (Anderson, 1988). Furthermore, anthropogenic activities such as tillage, fertilization, irrigation, and cultivation, etc., in soils exert considerable influence on the structure and functional performance of microbial communities via changing the soil properties, thus subsequently influence soil quality in the long term (Tripathi et al., 2015). To understand the diversity of microbial community and their function structure in soils, and their correlations with soil natural properties and human activities are therefore essential to evaluate the crops productivity and environmental sustainability of soil ecosystems, since microbes are responsible for the fertility and productivity of different soil types to a large degree (Anderson, 1988; Martiny et al., 2006).

Numerous recent studies based on culture-independent techniques have suggested that the diversity and community composition of soil microorganisms on large scales were greatly driven by soil pH, and some other soil properties such as organic matter and salinity (Lauber et al., 2009; Griffiths et al., 2011; Tripathi et al., 2015). These studies mainly concentrated on the microbial distribution pattern across large geographical distances, especially focusing on the surface soil, but rarely paid attention to subtle difference of microbial community among soil types. As soil type represents a consequence of the complex influences from soil parent materials and historical and present climatic conditions, it is difficult to attribute the influence of soil type on microbial community to a single factor (Cao et al., 2012; Zhao et al., 2016). For example, some studies suggested that certain microbial taxa would prefer to specific soil types, and soil bacterial community composition was distinct among soil types but could be hardly explained by a single soil chemical parameter (Nie et al., 2012; Tripathi et al., 2012). Moreover, it was observed that soils with different parent materials overwhelmingly supported distinct bacterial community structure after similar long term cultivation, as land use or management practices may mostly shift microbial community structures in top soils (Girvan et al., 2003; Sheng et al., 2015; Sun et al., 2015). Hence it is of interest to understand how much human disturbance could affect soil microbial community comparing to the effects from soil parent materials. Furthermore, the current knowledge on the differentiation of soil microorganism among various soil types and their potential significance are still very limited and deserved to be well depicted, considering the large body of pedodiversity and microbial diversity.

In addition to soil properties determined by soil parent materials, nutrient and oxygen fluctuating along soil profile depth can subsequently lead to a change in microbial communities with soil depth (Will et al., 2010; Wang et al., 2014; Stone et al., 2015). Compared to topsoil, sub-soil volume is much greater, and thus the microbial community and its function in sub-soil is not negligible. Some investigations focusing on specific microbes such as methane-oxidizing bacteria (Reim et al., 2012), ammonium oxidizing microbes (Wang et al., 2014; Lu et al., 2015), nitrifers and denitrifiers (Qin et al., 2016), and the rates of carbon and nitrogen cycling processes suggested great variation of functional microbes and the process they mediated along soil depth gradients (Durán et al., 2017), while few works have made efforts to compare the overall pattern of microbial functional genes in different soil depth layers. Paddy soils occupied large agricultural areas in China. Although the distribution of contrasting microbial community in different paddy soils and the functional analysis of microbes have been described recently (Sheng et al., 2015; Su et al., 2015), comparative investigations linked both taxonomic and functional structure are limited. Also, some recent studies attempted to exploring the influence of different land uses on both taxonomic and functional community of microbes combining high-throughput sequencing technique with functional gene array (Paula et al., 2014; Mendes et al., 2015), but these studies were mainly carried out in the same region within similar soil types.

Comparing to different management practices and land use types, soils with water-logged paddy rice cultivation receive relatively higher uniformity and similarity of management practice. It is still less understood whether uniform management would assimilate microbial community across different soil types, and the extent of inherent influences of parent materials on shaping microbial communities. Along a paddy soil profile, water would replace the gaseous phase and thus the oxygen status varies dramatically along the soil profile (Kögel-Knabner et al., 2010), but succession of microbial community structure along this oxygen gradient has rarely been studied (Noll et al., 2005). In our previous investigation, activity and diversity of the anaerobic ammonium oxidation (anammox) bacteria were examined in three paddy soil types, and the results revealed distinct pattern of anammox activity, diversity and abundance along depth gradient in different paddy soils (Bai et al., 2015). While the study only focused on the aspect of anammox process rather than a comprehensive observation on soil microbes and their functional genes. Therefore, in the present study, Miseq high-throughput sequencing and GeoChip techniques were combined to characterize the bacterial and archaeal community composition and functional structures across four soil profile depths among three distinct paddy soil types including Inceptisol, Oxisol, and Ultisol soil orders. This study aimed to (1) elaborately depict microbial community succession among different parent materials and explore the linkage between microbial community taxonomic and functional structure, and (2) to understand how much do soil inherent properties affect microbial community composition and its functional structure, and (3) if uniform flooding management would assimilate microbial community among different soil types.

## Materials and Methods

### Mesocosm Incubation and Soil Sampling

The paddy soils used for this study were freshly sampled from a greenhouse mesocosm incubation system as described in our previous study (Bai et al., 2015). Briefly, three paddy soils originally collected from Binhai (BH, 119.84° E, 34.01° N, Inceptisol), Leizhou (LZ, 110.04° E, 20.54° N, lateritic Oxisol), and Taoyuan (TY, 111.48° E, 28.90° N, Ultisol) in three rice production areas in Southeast China with the spatial distance more than 1000 km from each other, were incubated in mesocosms columns (50 cm in diameter and 70 cm in height) and received similar water, fertilization, and rice plantation management as in the field. For each soil type, two replicate columns were constructed, and one profile were sampled from each column at four soil depth intervals (A, 0–5 cm; B, 5–20 cm; C, 20–40 cm; D, 40–60 cm) at the tillering and heading growth stages of rice.

### Soil Physicochemical Determinations

Soil pH and EC were measured with a pH meter and a conductivity meter, respectively. NO_3_^-^ and NH_4_^+^ were extracted with 1 M KCl and determined by using a flow analyzer (AA3, SEAL analytical, Germany). Soil hot water-extractable carbon (HWC) was extracted with water at 70°C and determined by a carbon-nitrogen analyzer. Soil total C, N, and S were measured using an elemental analyzer (Vario EL III-Elementar, Germany). The concentrations of dissolved O_2_ along the soil profiles were also measured with an O_2_ microsensor electrode (Unisense, Denmark) precisely positioned by a micromanipulator (Unisense).

### DNA Extraction and Purification

The total genomic DNA of microbes was extracted from 5 g of dry soil by using a protocol that included liquid nitrogen grinding and sodium dodecyl sulfate as previously described (Zhang et al., 2013). To remove humus and protein components, the DNA was purified with 0.5% low melting point agarose gel, and further purified by a phenol-chloroform-butanol extraction procedure. DNA quantity and quality were evaluated using a NanoDrop ND-1000 Spectrophotometer (NanoDrop Technologies Inc., Wilmington, DE, USA), and all the DNA sample reached final A260/A280 and A260/A230 ratios of >1.7 and 1.8. All DNA samples were stored at -40°C before downstream analysis.

### DNA Microarray Hybridization, Scanning and Data Processing

Geochip 4.2 was utilized for analyzing functional structures of soil microbes. The experiments were carried out as previously described (Yang et al., 2013). Briefly, DNA samples were firstly labeled with Cy-5 fluorescent dye using a random priming method. Then the labeled DNA samples were purified with a QIA purification kit (Qiagen, Valencia, CA, USA) and further dried in a SpeedVac (ThermoSavant, Milford, MA, USA) at 45°C for 45 min. Hybridization buffer containing 40% formamide, 25% SSC, 1% SDS, 10 μg of unlabeled herring sperm DNA (Promega, Madison, WI, USA) was added to the dried DNA samples, then vortexed, spun down and incubated at 95°C for 5 min. Hybridizations were performed with a MAUI hybridization station (BioMicro, Salt Lake City, UT, USA). Subsequently, the microarray was scanned by a 100% laser power and 100% photomultiplier tube with a NimbleGen MS 200 Microarray Scanner (Roche, Madison, WI, USA) and signal intensities were quantified.

The obtained raw data was pre-analyzed and denoised with the online pipeline provided by Microarray Data manager^1^. Each sample was treated separately in this procedure. Briefly, values with signal-noise ratio less than 2 were firstly removed. Subsequently, the values of the signals detected by the probes within each sample were normalized with the lnMR method that ln(x+1) was divided by mean of total signal intensity of each sample, where x denotes a detected value by each probe within each sample.

### Illumina Mi-Seq Sequencing and Sequence Analysis

The V4–V5 region of bacterial and archaeal 16S rRNA genes were sequenced with primer sets 515F (5′-GTGCCAGCMGCCGCGGTAA-3′)/806R (5′-GGACTACHVGGGTWTCTAAT-3′) and Arch519F (5′-CAGCCGCCGCGGTAA-3′)/Arch 915R (5′-GTGCTCCCCCGCCAATTCCT-3′), respectively. Based on the requirement of Illumina sequencing, Illumina adaptor A was added to the 5′-ends, and Illumina adaptor B and barcode were added to the 3′-ends of primers. The raw sequencing data were further processed with QIIME software. The FLASH method was firstly carried out to assemble the obtained sequences, and then the UPARSE method was utilized to filter chemira and repetitive sequences. The OTUs of bacterial and archaeal sequences were defined according to a similarity of 97%, and OTU tables were established for further analysis. The identification of bacterial and archaeal taxa was conducted based on the Greengenes database.

### Statistical Analysis

Pearson correlation analysis and analysis of differences was carried out with Duncan analysis of one-way ANOVA by using SPSS software (IBM, version 19.0). The vegan package of R software (version 3.2.2) was utilized for conducting detrended correspondence analysis (DCA) which was used to study community and functional structures of soil microbes. Structural equation models (SEM) were developed according to an *a priori* model to determine the direct and indirect contribution of soil type, soil depth, pH, salinity, and HWC on microbial community and functional structures (as assessed by the first principal coordinate of the Bray–Curtis dissimilarity matrix). Soil type and depth were set as exogenous variables that latitude and longitude (first principal coordinate of the Bray–Curtis dissimilarity matrix) of the three soil sampling sites were substituted for soil type and soil depth was set as 5, 20, 40, and 60. SEM analysis was conducted by utilizing AMOS 22.0 (Amos, Development Corporation, Meadville, PA, USA). Adequate model fits were examined according to non-significant Chi-square test (*P* > 0.05), goodness fit index (GFI), Akaike value (AIC), and root mean square error of approximation (RMSEA).

### Construction of Phylogenetic Molecular Ecological Networks

Phylogenetic molecular ecological networks (pMENs) were constructed based on random matrix theory with the bacterial and archaeal OTUs obtained in profile A and D from the three paddy soils in this study (Deng et al., 2012). Network construction was carried out by utilizing the online pipeline provided by the Institute of Environmental Genomics, University of Oklahoma^2^.

The average connectivity index (avgK) and modularity index were utilized to describe the topology structure of the networks. The avgK was used to describe the complexity of the networks, and modularity was utilized as a measurement of system resistance. The topological roles of different nodes were divided into four sub-categories based on the within-module connectivity (Z_i_) and the among module connectivity (P_i_): (1) nodes with Z_i_ > 2.5 and P_i_ < 0.62 were defined as module hubs; (2) nodes with Z_i_ > 2.5 and P_i_ > 0.62 were defined as network hubs; (3) nodes with Z_i_ < 2.5 and P_i_ < 0.62 were defined as peripherals; (4) nodes with Z_i_ < 2.5 and P_i_ > 0.62 were defined as connectors (Olesen et al., 2006).

### Networks Analysis of Soil Microbial Community and Functional Genes

The associations of microbial taxa and functional genes were analyzed using the Cytoscape plug-in CoNet (Soffer et al., 2015). The detected taxa and genes with a minimum occurrence of four across all the samples within certain depth layer were discarded in prior to calculation in order to minimize the artificial association bias (Li et al., 2015). The pairwise calculation was performed at the phylum level for archaea and bacteria expect that *Proteobacteria* were analyzed on the level of class, while functional genes were employed according to the gene category provided by functional gene array (Bai et al., 2013). Combination of correlation scores and *P*-values of Spearman correlation, Pearson correlation, Kullback–Leibler dissimilarity, and Bray–Curtis dissimilarity were utilized for all the pairwise correlation. Potential false-positive correlations and compositionality biases were eliminated by ReBoot procedure with 100 permutations, and the resultant distribution was further refined with 100 bootstraps. Then *Brown* method was utilized to combine the *P*-values for the four correlations measurement, and correlations found to be significant by less than two methods were discarded (Soffer et al., 2015). Only correlations with a coefficient above 0.8 and a significance level below 0.05 were considered statistically robust which were finally displayed as previously reported (Hu et al., 2016). The obtained pairwise correlations were used to construct the co-association networks. Network topology was explored using Cytoscape software and the Network Analyzer plug-in and was illustrated on the open-source interactive platform Gephi (Bastian and Heymann, 2009).

## Results

### Soil Physical and Chemical Properties

Chemical properties of the three soil columns are shown in **Table 1**. Significant differences with respect to properties were observed between soil types and along profiles within each soil type. Soil water content decreased dramatically along soil profiles. The pH ranged from 8.0 to 8.6 in BH columns, ranged from 6.8 to 7.2 in LZ columns, and ranged from 5.8 to 6.0 in TY soils. Soil salinity which was reflected by soil electrical conductivity (EC) was approximately 10-fold lower in TY than in BH and LZ soils. HWC peaked in the first two soils layers of the three soil types, and was 2- to 8-folds higher in BH and TY soils than in the LZ soil. DCA for measured soil properties in the three paddy soils showed a clear division in soil characteristics among the three paddy soils. In addition, a succession of soil chemical parameters along soil depth was observed along soil depth within each soil type (Supplementary Figure 1).

**Table 1:** Physicochemical properties of soil samples from greenhouse mesocosm incubation^∗^.

Sample Name	Profile	HWC	pH	NH4	NO3	EC	OM
BH-A	0–5 cm	181.24 ± 20.89ˆb	7.96 ± 0.07ˆc	15.58 ± 7.19ˆa	0.7 ± 0.77ˆb	0.33 ± 0.13ˆab	2.36 ± 0.27ˆcde
BH-B	5–20 cm	155.98 ± 16.23ˆbc	8.23 ± 0.02ˆb	7 ± 3.66ˆb	0.18 ± 0.13ˆb	0.38 ± 0.09ˆa	2.86 ± 0.13ˆb
BH-C	20–40 cm	88.43 ± 26.11ˆcd	8.51 ± 0.07ˆa	7.75 ± 2.14ˆb	0.2 ± 0.2ˆb	0.29 ± 0.03ˆab	1.63 ± 0.37ˆf
BH-D	40–60 cm	62.13 ± 14.3ˆd	8.62 ± 0.04ˆa	6.65 ± 0.89ˆb	0.21 ± 0.18ˆb	0.26 ± 0.02ˆab	1.48 ± 0.27ˆf
LZ-A	0–5 cm	63.33 ± 40.85ˆd	7.02 ± 0.21ˆde	5.81 ± 3.78ˆb	1.28 ± 1.38ˆab	0.35 ± 0.2ˆab	2.61 ± 0.07ˆbc
LZ-B	5–20 cm	51.6 ± 18.62ˆd	6.79 ± 0.09ˆf	7.54 ± 3.98ˆb	1.17 ± 1.18ˆab	0.25 ± 0.03ˆab	2.5 ± 0.21ˆcd
LZ-C	20–40 cm	45.93 ± 6.95ˆd	6.85 ± 0.14ˆef	9.36 ± 2.75ˆab	0.9 ± 0.82ˆab	0.23 ± 0.03ˆb	2.32 ± 0.17ˆcde
LZ-D	40–60 cm	63.79 ± 26.6ˆd	7.15 ± 0.23ˆd	7.48 ± 4.33ˆb	1.11 ± 1.22ˆab	0.25 ± 0.05ˆab	2.49 ± 0.22ˆcd
TY-A	0–5 cm	409.93 ± 139.76ˆa	5.96 ± 0.21ˆgh	9.25 ± 5.97ˆab	2.6 ± 2.83ˆa	0.06 ± 0.05ˆc	3.74 ± 0.17ˆa
TY-B	5–20 cm	402.87 ± 40.98ˆa	5.81 ± 0.13ˆh	9.35 ± 1.6ˆab	0.79 ± 0.63ˆab	0.03 ± 0.02ˆc	3.51 ± 0.08ˆa
TY-C	20–40 cm	117.03 ± 31.37ˆbcd	6.09 ± 0.1ˆg	8.61 ± 4.59ˆb	0.76 ± 0.5ˆab	0.02 ± 0.01ˆc	2.27 ± 0.10ˆde
TY-D	40–60 cm	110.2 ± 27.61ˆbcd	6.04 ± 0.09ˆg	9.24 ± 3.5ˆab	0.8 ± 0.45ˆab	0.02 ± 0.01ˆc	2.15 ± 0.11ˆe

**Sample Name**	**Profile**	**AOFe**	**DCBFe**	**S**	**interMn**	**reducMn**	**Water content**

BH-A	0–5 cm	1.24 ± 0.12ˆd	4.66 ± 0.11ˆd	0.031 ± 0.004ˆd	0.12 ± 0.011ˆc	0.0067 ± 0.0005ˆa	1.08 ± 0.42ˆb
BH-B	5–20 cm	1.55 ± 0.38ˆc	5.19 ± 0.61ˆd	0.032 ± 0.002ˆd	0.12 ± 0.004ˆc	0.0068 ± 0.0005ˆa	0.5 ± 0.04ˆc
BH-C	20–40 cm	0.84 ± 0.11ˆe	4.38 ± 0.12ˆd	0.028 ± 0.004ˆd	0.15 ± 0.008ˆc	0.0057 ± 0.0007ˆb	0.33 ± 0.01ˆc
BH-D	40–60 cm	0.68 ± 0.04ˆe	4.2 ± 0.06ˆd	0.024 ± 0.002ˆd	0.16 ± 0.01ˆc	0.0053 ± 0.0006ˆb	0.34 ± 0.01ˆc
LZ-A	0–5 cm	2.6 ± 0.07ˆa	121.63 ± 4.51ˆa	0.043 ± 0.005ˆa	0.31 ± 0.032ˆa	0.0051 ± 0.0005ˆb	1.2 ± 0.25ˆb
LZ-B	5–20 cm	2.34 ± 0.07ˆb	123.63 ± 8.99ˆa	0.048 ± 0.003ˆa	0.24 ± 0.016ˆb	0.0036 ± 0.0005ˆc	0.57 ± 0.01ˆc
LZ-C	20–40 cm	2.34 ± 0.12ˆb	118.31 ± 1.75ˆa	0.043 ± 0.003ˆa	0.26 ± 0.032ˆab	0.0041 ± 0.0003ˆc	0.52 ± 0.01ˆc
LZ-D	40–60 cm	2.28 ± 0.07ˆb	123.73 ± 10.26ˆa	0.041 ± 0.006ˆa	0.31 ± 0.12ˆa	0.0051 ± 0.0006ˆb	0.49 ± 0.04ˆc
TY-A	0–5 cm	1.28 ± 0.05ˆd	19.04 ± 0.5ˆc	0.039 ± 0.001ˆc	0.026 ± 0.004ˆd	0.0014 ± 0.0001ˆd	2.18 ± 0.81ˆa
TY-B	5–20 cm	1.15 ± 0.08ˆd	18.63 ± 0.77ˆc	0.037 ± 0.003ˆc	0.021 ± 0.001ˆd	0.0014 ± 0.0002ˆd	0.58 ± 0.02ˆc
TY-C	20–40 cm	2.48 ± 0.14ˆab	30.27 ± 0.93ˆb	0.022 ± 0.002ˆb	0.046 ± 0.002ˆd	0.0039 ± 0.0003ˆc	0.38 ± 0.03ˆc
TY-D	40–60 cm	2.41 ± 0.06ˆab	29.99 ± 0.59ˆb	0.021 ± 0.002ˆb	0.045 ± 0001ˆd	0.0036 ± 0.0005ˆc	0.41 ± 0.02ˆc

*^∗^A, B, C, and D represent soil sampling layers 0–5 cm, 5–20 cm, 20–40 cm, and 40–60 cm (the same below); HWC, hot water-extractable organic carbon (mg kg^-*1*^); NH4, NH_4_^+^concentration (mg kg^-*1*^); NO3, NO_3_^-^ concentration (mg kg^-*1*^); EC, electrical conductivity (d Sm^-*1*^); OM, organic matter (g kg^-*1*^); N, total nitrogen (%); C, total carbon (%); S, total sulfur (%); C/N, carbon to nitrogen ratio; AO-Fe, ammonium oxalate extractable Fe (mg kg^-*1*^); DCB-Fe, DCB extractable Fe (mg kg^-*1*^); interMn, interchangeable Mn (mg kg^-*1*^); reducMn, reduceable Mn (mg kg^-*1*^); Water content, water content*.

*The different letters indicate significant differences of measured values within all the samples by one-way ANOVA*.

### Diversity Based on the 16S rDNA and Microarray Analysis

A total of 48 samples covering 3 soil types, 4 depth layers, 2 mesocosm replicates, 2 growth stages were subjected to MiSeq sequencing and microarray assay to explore the diversity of species and functional genes. A total of 26075 bacterial OTUs and 3404 archaeal OTUs were generated after resampling with 42364 bacterial reads and 9456 archaeal reads per sample, respectively. No significant difference was found in alpha diversity of bacterial community except sample LZ-A presented the lowest Shannon indices. While Shannon indices of archaeal community was the lowest in layer A of BH and TY soils, and the highest and lowest Simpson indices were detected in TY-C and LZ-D, respectively (**Table 2**).

**Table 2:** Number of observed species, indices of Shannon and Simpson diversity of bacterial and archaeal community in four soil profiles of three paddy soils.

	Bacteria			Archaea		
Sample	Observed species	Shannon	Simpson	Observed species	Shannon	Simpson
BH-A	2906	10.32 ab	1.00 a	61	2.72 e	0.74 de
BH-B	3834	10.99 a	1.00 a	294	4.23 bc	0.84 bc
BH-C	3965	10.97 a	1.00 a	319	4.99 a	0.91 ab
BH-D	3644	10.76 a	1.00 a	315	5.11 a	0.92 ab
LZ-A	2518	8.42 b	0.90 a	125	3.88 cd	0.86 abc
LZ-B	2672	9.86 ab	1.00 a	152	3.84 cd	0.84 bc
LZ-C	2633	9.86 ab	1.00 a	161	4.03 cd	0.84 abc
LZ-D	2643	9.91 ab	1.00 a	144	3.55 d	0.79 d
TY-A	2985	9.28 ab	0.93 a	192	3.46 d	0.66 cd
TY-B	3106	10.16 ab	0.99 a	222	4.70 ab	0.90 ab
TY-C	2668	9.96 ab	1.00 a	232	5.00 a	0.93 a
TY-D	2507	9.57 ab	0.99 a	207	4.84 ab	0.92 ab

*The different letters indicate significant differences of measured values within all the samples by one-way ANOVA*.

The number of detected genes, Shannon index, inverse Simpson index and Simpson evenness by Geochip were calculated to evaluate the functional diversity and structure of microbial communities. In total, 44350 genes were detected and ranged from 36860 to 40612 in different samples. Shannon and Simpson diversity in LZ samples were significantly lower than in BH and TY samples, and showed no significant difference between BH and TY samples. Approximately 80% of functional genes were shared by three soil types while around 5% of the genes were unique within each soil type (**Table 3**).

**Table 3:** Shared and endemic genes among soil types, and diversity indice of functional genes.

Sample name	BH	LZ	TY
BH	**2264 (5.57%)**	*34397 (79.85%)*	*37842 (87.58%)*
LZ		**1141 (3.10%)**	*35213 (83.67%)*
TY			**1275 (3.15%)**
Genes detected	40612	36860	40439
Shannon index	10.36 a	10.22 b	10.34 a
Inverse Simpson index	31536.83 a	27739.00 b	31171.78 a

*Bold numbers denote the number and percentage of endemic genes within each soil types, while italic numbers denote the number and percentage of shared genes between soil types*.

*The different letters indicate significant differences of measured values within all the samples by one-way ANOVA*.

### Community Similarity Based on the 16S rDNA and Microarray Analyses

**FIGURE 1:**
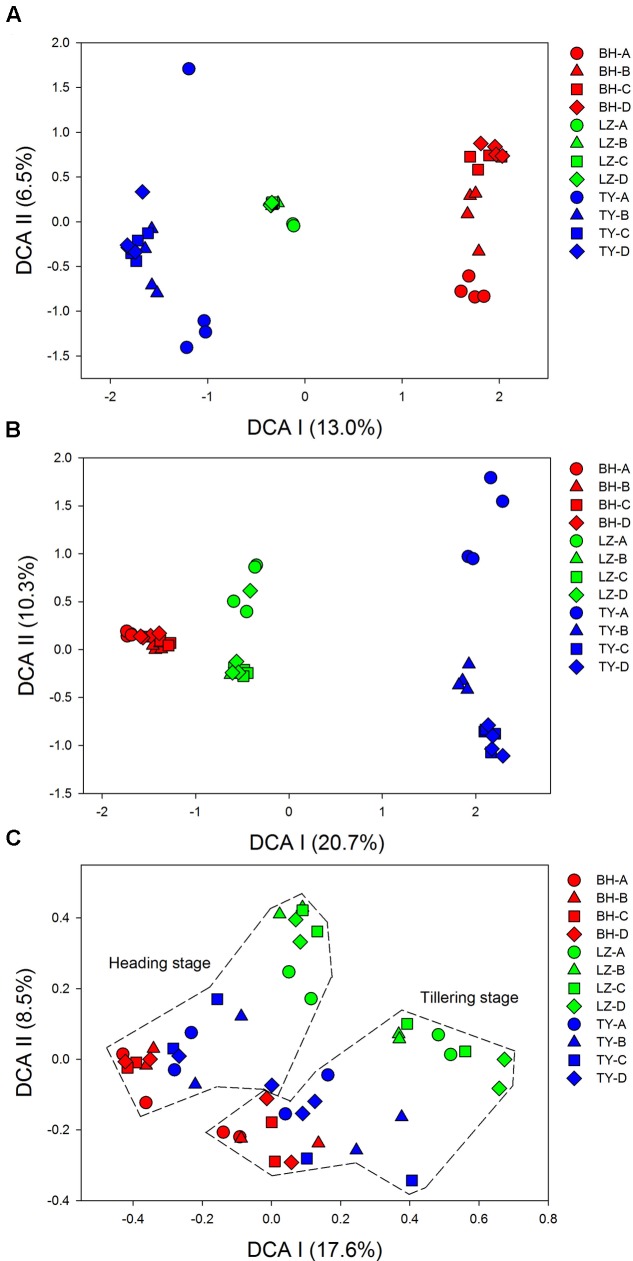
Detrended correspondence analysis (DCA) for microbial taxonomic and functional community. Results of **(A)** bacterial community, **(B)** archaeal community, and **(C)** all of the functional genes from four soil profiles (A, B, C, D) of three paddy soils (BH, LZ, TY).

Detrended correspondence analysis analysis of 16S rDNA Mi-seq sequences data indicated that bacterial and archaeal communities were clearly separated into three groups according to soil types, indicating the effects of soil type on microbial community composition (**Figures 1A,B**). A clear trend of community succession along soil profiles was also observed in the three soil types, with surface soils (0–5 cm) separated from sub-soils (5–20 cm, 20–40 cm, and 40–60 cm) markedly, and community structure in surface soils was much more heterogeneous compared to deeper soil layers (Supplementary Figure 2). Given the relative low variation in bacterial and archaeal community structure at the two paddy rice growth stages, samples within the same depth of each soil at the two time points were considered as replicates in the following analysis for the 16S rDNA data.

Detrended correspondence analysis based on functional gene array data showed that samples from BH, LZ, and TY soils were well separated from each other by DCA I and DCA II, and the distance between BH and TY samples was closer than to LZ samples (**Figure 1C**). Unlike community pattern based on 16S rDNA, however, there was no clear differentiation in the structure of functional community between profile layers within each of the soil types. By contrast, samples of tillering and heading stages formed two clusters, which implied the significant difference of soil microbial functional structure between the two growth stages of paddy rice (**Figure 1C** and Supplementary Figure 2).

### Phylogenetic Molecular Network Analysis of Microbial Communities

**FIGURE 2:**
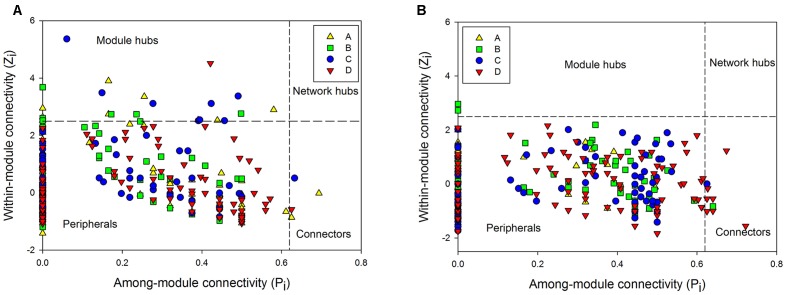
The Z-P plot indicating categories of nodes according to Z_i_ (within-module connectivity) and P_i_ (among-module connectivity) of bacterial **(A)** and archaeal community **(B)** in four soil profile layers.

To further understand the similarity of microbial communities, networks of bacteria and archaea from the three soil profiles were constructed with OTU data obtained from Mi-seq sequencing (**Table 4**). In respect of the bacterial networks, the network size indicated by the numbers of nodes was highest (206) in bacteria community from layer B (**Table 4**). The coefficient of positive correlation among bacterial community in layer A was up to 93%, and 18–27% higher than in other layers. However, the network for bacteria in layer A had the lowest average degree (avgK = 3.68), which suggested higher connectivity of the nodes in deeper soil layers. Also, bacterial community in layer A presented the highest level of modularity, indicated its higher system resistance to changes in comparison with other layers. Meanwhile, module hubs, representing key nodes species in the networks, were detected in all the layers. Surface soil harbored the most module hubs (nine hubs) among the soil layers and layer B and C harbored six and eight hubs, while only one hub was defined in the bottom soils, suggesting a lower amount of generalization in this soil depth (**Figure 2A** and **Table 4**).

**Table 4:** Major topological properties of phylogenetic molecular ecological networks of bacterial and archaeal communities in four profile layers (A to D) of the three paddy soils.

Community	Similarity threshold	Network size	Total links	No. of module hubs	Percentage of positive links	Percentage of negative links	avgK	Modularity
Bacteria (A)	0.82	191	351	9	93%	7%	3.68	0.68
Bacteria (B)	0.85	166	452	6	74%	26%	5.45	0.63
Bacteria (C)	0.83	206	527	8	66%	34%	5.12	0.61
Bacteria (D)	0.83	159	478	1	75%	25%	6.01	0.54
Archaea (A)	0.76	51	156	0	63%	37%	6.12	0.45
Archaea (B)	0.75	77	344	2	61%	39%	8.94	0.38
Archaea (C)	0.77	84	446	0	58%	42%	10.62	0.31
Archaea (D)	0.74	103	479	0	74%	26%	9.30	0.41

Depth-effects on the connection among microbes were also observed in the networks of the archaeal community. Similar to bacteria, the lowest avgK (6.12) but highest modularity (0.45) in the networks of archaeal community were recorded in layer A (**Table 4**), indicating that archaea had a lower connectivity in surface soils than in deeper soil layers and possessed a higher system resistance in comparison with other profile depths. In contrast to bacterial networks, module hub was hardly detected in all the archaeal networks (**Table 4**), suggesting the lack of generality in the archaeal community in these soils. However, more connector nodes between modules were identified in the archaeal community, especially in the bottom layer (**Figure 2B**). Although there was a slight decrease in the coefficient of positive correlation among archaeal nodes from profiles A to C, the highest coefficient was detected in the bottom layer of the archaeal community (**Table 4**).

### Bacterial and Archaeal Community Composition Based on Mi-Seq Sequencing

**FIGURE 3:**
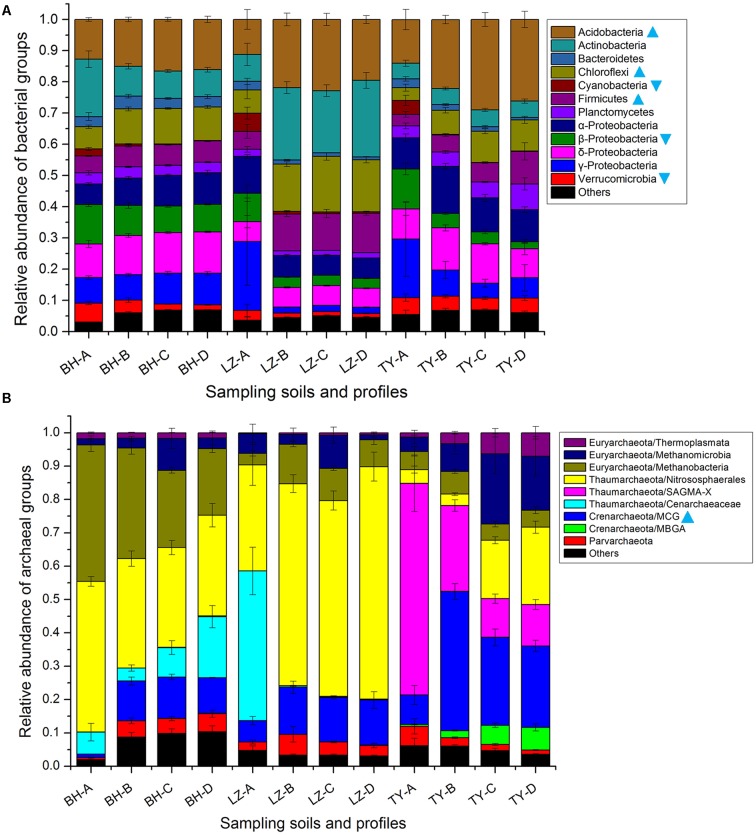
Dominant taxonomic groups of bacterial **(A)** and archaeal **(B)** groups in four soil profiles of three paddy soils. Cyan triangles denote that the relative abundance of the group uniformly decreased along depth layers within each soil type, cyan inverted triangles denote that the relative abundance of the group uniformly increased along depth layers.

Totally, 34 bacteria phyla were identified in all 48 samples of three soil types. The dominant phyla, including *Acidobacteria*, *Actinobacteria*, *Bacteroidetes*, *Chloroflexi*, *Cyanobacteria*, *Firmicutes*, *Planctomycetes*, *Proteobacteria*, and *Verrucomicrobia*, accounted for more than 95% of bacterial sequences (**Figure 3A**). The relative abundance of individual bacterial taxa varied distinctly among different paddy soil types and between surface and subsurface layers. Of the eight abundant bacteria phyla, the relative abundance of *Acidobacteria*, *Chloroflexi*, and *Firmicutes* were the lowest in the three surface soils (11.2–14%, 3.5–7.4%, and 3.6–5.8%, respectively) but were relatively higher in all the deep soil layers (11.2–29%, 7.7–17.8%, and 5.5–12.6%, respectively), and positively correlated with profile depth (*P* < 0.01, *n* = 48, Supplementary Table 1), also negatively correlated to soil HWC (*P* < 0.01, *n* = 48, Supplementary Table 1). Conversely, the abundance of *Verrucomicrobia* and β*-proteobacteria* decreased along soil depth in all the soil profiles and both positively correlated to the fluctuation of soil HWC along depth (*P* < 0.01 and *P* < 0.05, *n* = 48, Supplementary Tables 1, 2). *Cyanobacteria* mainly presented in surface soil layer with a proportion of 2.2–5.8% rather than 0.2–0.9% in deeper soils (**Figure 3A**).

As for archaea, total 3404 OTUs were retrieved and classified into four phyla, including *Thaumarchaeota*, *Euryarchaeota, Crenarchaeota*, and *Parvarchaeota* (**Figure 3B**). The *Thaumarchaeota*, which composed of *Nitrososphaerales*, *Cenarchaeaceae*, and SAGMA-X, was the most abundant phylum and predominated in the surface layers of three soil types with a relative abundance of 51.8–76.7%, but accounted for a relatively lower proportion between 29.1 and 70% in subsurface layers of three soil types (**Figure 3B**). *Nitrososphaerales* accounted for 30–45.1%, and 31.8–69.7% of *Thaumarchaeota*-affiliating sequences in BH and LZ profile soils, respectively, while it only accounted for 3.3–23.2% in the TY profile soil (**Figure 3B**). *Euryarchaeota* was the second abundant archaeal phylum and mainly composed of classes *Methanobacteria*, *Methanomicrobia*, and *Thermoplasmata*. *Methanobacteria* was approximately 2–3 times higher in BH soils than in LZ and TY soils, *Methanomicrobia* and *Thermoplasmata* were more frequently detected in the sub layers of the TY soil (**Figure 3B**). Converse to *Thaumarchaeota* phylum, the phylum *Crenarchaeota* accounted for a much lower proportion in surface layer than deep layers in three soil types, and was more abundant in TY deep layers than in corresponding layers of BH and LZ columns (**Figure 3B**). The phylum *Crenarchaeota* was composed of MCG group and MBGA group. MBGA group was only detected in TY profiles with an increasing trend from 3 to 56% along the depths (**Figure 3B**), while MCG group occupied much lower abundance (1–8.9%) in surface soils than in sub-soils (10.7–41.7%) in all three soil types. In contrast, the group SAGMA-X of *Thaumarchaeota* was predominant in the TY surface soil (63.4%) but sharply decreased in deep layers (11.5–25.8%), while only occupied 0.01–0.06% proportion of BH and LZ archaeal communities (**Figure 3B**). All these further suggested distinct bacterial and archaeal community composition among the three soil types and the depth-dependent distribution pattern of some microbial groups.

### Pattern of Functional Gene Categories

**FIGURE 4:**
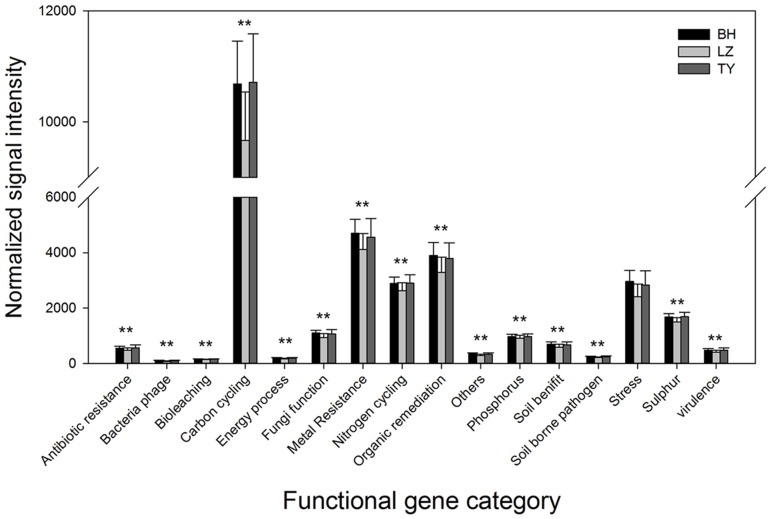
Abundance of different of categories of microbial functional genes in three paddy soils. ^∗∗^ Denotes that the abundance is significantly higher in BH and TY than in LZ soils.

Microbial functional genes were categorized based on the major metabolic processes to understand the pattern of functional microbial communities in different soil types and depth layers. Genes involved in carbon cycling were most abundant in all the samples, followed by genes related to metal resistance, organic remediation, and nitrogen cycling which also presented at a high level (**Figure 4**). DCA analysis did not identify significant differences in the gene abundance of different functional categories among depth layers within each soil type (data not shown). Normalized gene signal intensity of all the functional gene categories from different soil layers within each column were therefore combined. Results from one-way ANOVA indicated that the normalized signal intensity of most of the gene categories in BH and TY columns were significantly higher than in LZ columns (**Figure 4**).

### Linkage among Microbial Community, Functional Community, and Soil Properties

**FIGURE 5:**
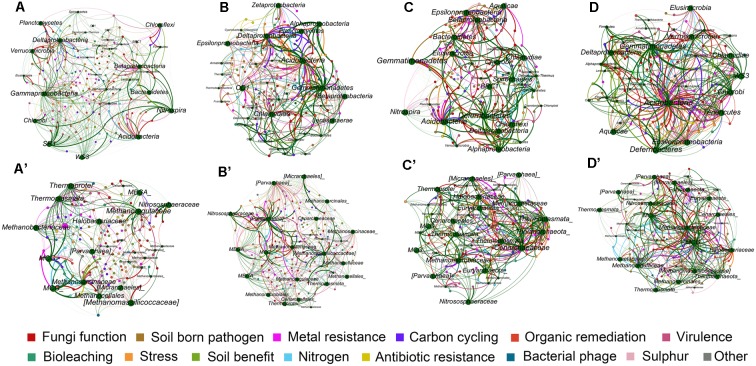
Mutualistic networks of interaction between bacterial community and functions of the four soil profile layers **(A–D)**, and archaeal community and functions **(A′–D′)**. Edges in green colors represent influence of microbes to ecological functions, and edges in other colors represent influence of each functional category to microbes.

Network analysis of microbial taxa and functional genes were generated to estimate the potential linkages between the microbial taxonomic community and overall functional structure obtained from GeoChip analysis (**Figure 5**). The average degree of the networks indicated the amount of total connections between microbes and their functions. For bacterial community, the highest average degree was detected in layer A (2.79), while archaeal community had lowest average degree in layer A (2.09) but relatively higher degree in both layers B and D (**Table 5**), indicating that bacterial community had complex connection with their functions in surface soil while archaea performed complex connections in deeper soils.

**Table 5:** Major topological properties of mutualistic networks between archaeal communities and functions, and between bacterial communities and functions from profiles A to D of the three paddy soils.

Community	No. of nodes	No. of edges	Average degree	Modularity
Bacteria (A)	194	541	2.79	0.57
Bacteria (B)	194	436	2.25	0.45
Bacteria (C)	179	413	2.31	0.53
Bacteria (D)	184	466	2.53	0.44
Archaea (A)	163	353	2.09	0.55
Archaea (B)	186	528	2.84	0.48
Archaea (C)	182	396	2.18	0.49
Archaea (D)	185	504	2.72	0.43

The number of edges that linked microbial taxonomic nodes with functional nodes reflects the linkage between microbial groups and functions, and the thickness of the edges showed the intensity of connection between nodes. Although layers B, C, and D of bacterial networks, and layers A and C of archaea presented overall lower average degrees (**Figure 5** and **Table 5**), stronger connections indicated by number of edges between nodes were observed in these networks (Supplementary Table 3). Generally, the numbers of edges of the main dominant bacterial phyla detected by sequencing were similar among the profile layers (**Figure 5** and **Table 5**). Specifically, *Acidobacteria* had relatively strong connections to ecological functions across all soil profile layers, while intensive connections between functions and some microbial groups such as δ-*Proteobacteria*, γ-*Proteobacteria*, *Planctomycetes*, and *Nitrospira* peaked in surface soils or upper layers (Supplementary Table 3). Most of the archaea groups presented weak connection with functions in the surface soils, while *Methanomicrobia* and *Parvarchaeota* in layers B and D, and *Methanobacteria* and MCG in layer D had strong connections with functions, and occupied nearly half of the archaeal connections in the two layers (Supplementary Table 4).

**FIGURE 6:**
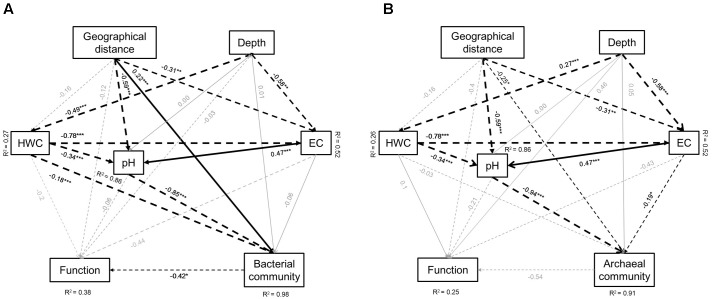
Effects of soil type (geographic distance), depth, soil HWC, pH and salinity (EC) on bacterial community and functional **(A)** and archaeal community and functional **(B)** structure. Geographical distance includes longitude and latitude of the soil sampling sites. Solid lines denote positive effects, and broken lines denote negative effects. Thickness of the arrows denotes significance and strength of the influence. R^2^ represent percentage of explanation of the models on the chosen factors. Significant level: ^∗^*P* < 0.05, ^∗∗^*P* < 0.01, ^∗∗∗^*P* < 0.001. Goodness-of-fit statistics are as following: **(A)** Chi-square = 0.000, degrees of freedom = 1, RMSEA = 0.000, AIC = 54, GFI = 1.000; **(B)** Chi-square = 0.000, degrees of freedom = 1, RMSEA = 0.000, AIC = 54, GFI = 1.000.

We further developed structure equation models (SEM) to explore how microbial communities and function associated. Parameters in the models included geographical distance of the soil originally located, soil depth, HWC, pH, and EC. The model for bacteria explained 98 and 38% of the variation in bacterial community and functional structure, respectively, along four depth layers of three paddy soils (**Figure 6A**). Soil pH, HWC, and geographical location were identified as the significant factors that shape the bacterial community structure, while microbial functional structure was solely affected by bacterial community (**Figure 6A**). Standardized total effects (including direct and indirect effects) obtained from standardized SEM indicated that soil pH contributed more to bacterial community structure than geographical distance and HWC (Supplementary Figure 3A). The model for archaea explained 91 and 25% of the variation in archaeal community and microbial functions, respectively. Archaeal community was mainly influenced by spatial distance, soil pH and EC (**Figure 6B**). Similar to bacteria, standardized SEM revealed that pH had stronger impact on archaeal community structure than geographical distance and EC (Supplementary Figure 3B).

## Discussion

### Distinct Differentiation of Microbial Community Structure Within Soil Types and Depth Layers Revealed by 16S rDNA and Network Analysis

One of the main purposes of this study was to reveal the effects of soil parent material and profile depth on both microbial community and functional structures. In particular, Illumina Mi-Seq sequencing combining GeoChip techniques and further network analysis facilitated the work and revealed manifest differentiation of bacterial and archaeal community structure among three paddy soil types and between surface and deeper soils within each soil type. Some previous investigations have concerned the microbial community characteristics across different sampling sites and depths and found that layer depths had overwhelming influence on microbial community structure than sampling location (Fierer et al., 2003; Hartmann et al., 2009; Eilers et al., 2012; Steven et al., 2013). However, these studies were mainly carried out in sites with similar soil properties. The three soil types tested in the present study represented distinct pedogenic history and therein possessed varied soil chemical properties. Further SEM analysis identified soil pH, HWC and salinity as the critical influencing factors significantly influencing microbial community structure (**Figure 6**). Specifically, soil types had a greater direct effect on pH (*P* < 0.001) comparing to soil salinity (*P* < 0.01) and HWC (*P* > 0.05) while pH was also significantly adjusted by HWC and salinity, and pH had stronger impact on bacterial and archaeal community than HWC and salinity in SEMs (**Figure 6** and Supplementary Figure S3). Generally, pH is the most representative characteristics of various parent materials of the soil chemical parameters (Imaya et al., 2005). Accordingly, it is not surprising to see that soil parent materials significantly affect microbial community structure via the determination of soil pH in this study. It either well explained why the observed variation of bacterial and archaeal community structure in this study were less among profile depths than among soil types, since the fluctuation of pH was smaller along profiles than that among soil types (7.96–8.62 in BH profile; 6.79–7.15 in LZ profile; 5.96–6.09 in TY profile). The results were consistent with numerous previous studies showing that soil pH is a critical factor in determining soil microbial community structure on large scale (Lauber et al., 2009; Griffiths et al., 2011; Kuramae et al., 2012; Thomson et al., 2015; Tripathi et al., 2015).

Furthermore, although the main division was caused preferentially by soil types, bacterial and archaeal community structure significantly changed along the four profile depths in this study. Particularly, a large separation of microbial community between surface (0–5 cm) and deep layers (5–60 cm) within each soil was indicated in DCA analysis (**Figure 2** and Supplementary Figure S1), which was most likely caused by more uniform physicochemical characteristics shared by deeper soil layers (Eilers et al., 2012). SEM analysis clearly showed that soil HWC was greatly driven by depth and significantly affected bacterial community in this study (**Figure 6A**). Similarly, Hartmann et al. (2009) also suggested that the availability of resources is one of the main factors determining soil microbial community composition along depth gradients, and copiotrophic microbes favored surface soils while oligotrophs preferred deeper soils. These results further revealed a nutrient-effects on microbial community along soil depth layers. In addition to pH and HWC, many previous studies also suggested that salinity has considerable impacts on activity, biomass and community structure of soil microbes (Asghar et al., 2012; Yan and Marschner, 2013; Teng et al., 2014; Morrissey and Franklin, 2015). In relation to that, our SEMs indicated archaeal community structure was significantly shaped by soil salinity while salinity was significantly influenced by both soil types and depth gradients (**Figure 6B**). Such pattern implied parent material and depth determined archaeal status via the changing of salinity in these soils.

Network analysis of 16S rDNA data further suggested depth-effects on the connection among microbes. Firstly, the modularity of bacterial and archaeal communities was the highest in the top soils and decreased with soil depth (**Table 4**), indicating a higher degree of habitat heterogeneity for microbes in upper soils (Wang et al., 2015). Also, modularity could serve as an indicator of system resistance (Carpenter et al., 2012). Therefore, the highest modularity of bacterial and archaeal network in top layers was suggestive of a relatively higher system resistance to changes compared to networks in deeper soil layers. Similarly, the lowest avgK value of the network in top layers indicated a low intensive interaction within bacteria and archaea (**Table 4**). Consequently, the higher modularity and lower avgK value in top layers both suggested that the bacterial and archaeal community in the surface soils would be more resistant and less influenced by disturbance than deeper soils according to network theory (Saavedra et al., 2011). Interestingly, despite bacterial and archaeal community had similar patterns of modularity along soil depth, the higher percentages of negative links within the bacterial community in layers B, C, and D were observed, while the higher proportions of negative links within archaeal community were detected in upper soils (**Table 4**), suggesting the competition on resources within bacterial and archaeal community was fiercer in deeper and surface soils, respectively. Such a phenomenon might imply a division of positive interactions of the two kingdoms within soils and archaea might be more adapted to the extreme environments in deeper soils.

### Variation of Main Bacterial and Archaeal Groups in Different Soil Types and Depth Layers Revealed by 16S rDNA Analysis

The microbial taxonomy analysis based on 16S rRNA gene suggested that *Acidobacteria*, *Actinobacteria*, *Proteobacteria*, *Chloroflexi*, *Firmicutes*, and *Verrucomicrobia* occupied approximately 80–90% of the bacterial communities in the three soil types, and some microbial taxa showed specific distribution patterns according to soil types or profiles. For instance, the highest relative abundance of *Acidobacteria* was recorded in the acidic TY Ultisols (14–29%), while the lowest was in the alkaline BH Inceptisols (12.7–16.6%). Pearson correlation analysis further proved that the relative abundance of *Acidobacteria* negatively correlated with soil pH (*P* < 0.01, *r* = -0.48, *n* = 48). The results were consistent with the previous studies’ conclusions that *Acidobacteria* are sensitive to pH change and this phylum thrives in relatively acidic soils (Will et al., 2010; Mendes et al., 2015).

Except to the bacterial groups such as *Planctomycetes*, α-*Proteobacteria*, and δ-*Proteobacteria* evenly distributed in each layer, the other groups showed consistently preference to a certain depth layer in the three soil types. Particularly, *Cyanobacteria, Verrucomicrobia*, and β-*proteobacteria*, were more abundant in all surface samples than in any other sub-soils. *Cyanobacteria* presented in the surface abundantly but hardly detected in sub-soils, which could be explained by their phototrophic metabolic characteristic. A higher relative abundance of β-*proteobacteria* was also reported in an early study in the oxic zone of paddy soils (Lüdemann et al., 2000), indicated the class prefers a relatively oxygen-rich condition. The highest relative abundance of *Verrucomicrobia* were observed in surface soils for three soil types in the present study. Similarly, *Verrucomicrobia* was found to peak at middle depth layers (20–40 cm) in upland soils and it was suggested that *Verrucomicrobia* might prefer a relative anoxic circumstance rather than oxic and extreme anoxic conditions in previous studies (Hansel et al., 2008; Eilers et al., 2012). For the paddy soils used in this study, the surface samples at two sampling points were flooded or at least water-saturated and therein represented an oxic/anoxic interface. The highest relative abundance of *Verrucomicrobia* in this layer and decreasing trend along profile depth further suggested its preference to such oxic/anoxic habitat.

By contrast, *Firmicutes*, *Chloroflexi*, and *Acidobacteria* were more abundant in sub-soils than in surfaces in all three soil types. Increasing of *Firmicutes* along soil profiles was observed in all three soil types, which is consistent with the observations in previous studies (Hansel et al., 2008; Li et al., 2014), and could be explained by their adaptation to low-nutrient environments in deeper soil layers as *Firmicutes* usually thrive in extreme conditions through spore-forming (Li et al., 2014). The decline tendency of *Chloroflexi* and *Acidobacteria* along soil depth coincided with a recent study in a colluvial soil in which the relative abundance of two phyla decreased along the profile up to 80 cm depth but fluctuated in deeper soils of 80–380 cm (Sagova-Mareckova et al., 2016). Moreover, the strong inverse relationship between carbon availability and the abundance of *Acidobacteria* were frequently observed in various soil systems and *Acidobacteria* was supposed to be “oligotrophic” (Fierer et al., 2007; Hansel et al., 2008). The clear decrease in abundance of *Acidobacteria* in carbon-poor deep profiles observed in this study further supported this hypothesis. Besides these groups showing consistent trend in three soil types, some predominant bacterial phyla such as α-, γ-*Proteobacteria*, and *Actinobacteria* did not show consistent shifts in relative abundance along the profiles of three soil types, suggesting the specific microbial community characteristics in different soil types (Eilers et al., 2012).

Taxonomy results of archaeal 16S rRNA gene reads showed that *Thaumarchaeota* was the most predominant phyla (36.7–76.7%) across each layer and three soil types in this study, while *Crenarchaeota* only accounted for 1.03–43.8% of archaeal reads, which was consistent with the observation that *Thaumarchaeota* dominated archaeal community in soil systems (Hu et al., 2013; Chronakova et al., 2015; Schneider et al., 2015; Tripathi et al., 2015). Generally, *Thaumarchaeota* was more abundant in all surface soils, and such a pattern was similar to a previous finding that *Thaumarchaeota* was the only archaeal group in aerobic top soils, in contrast to the more diverse archaeal community in deeper soil layers (Mikkonen et al., 2014). Likewise, proteins from *Thaumarchaeota* were mainly found in relatively oxic environments along a gradient of oxygen and redox in oxygen minimum zones (Hawley et al., 2014), suggesting the distribution of *Thaumarchaeota* was probably oxygen dependent. It was not surprise as the archaeal ammonia oxidizers are mainly affiliated within this phylum. Specifically, *Nitrososphaerales* within the phylum *Thaumarchaeota* was widely detected, with high relative abundance especially in all profile layers of BH and LZ soils, suggesting high abundance of ammonia oxidizers. Interestingly, *Cenarchaeaceae* and SAGMA-X within *Thaumarchaeota* showed contrasting distribution patterns across soil samples in the present study. *Cenarchaeaceae* was only found in BH soils and top soils of LZ columns (6.6–44.8%) while SAGMA-X was mainly detected in TY profiles and predominated in surface of TY soils (63.4%) (**Figure 3**). However, these two groups were rarely recorded in previous reports except that *Cenarchaeaceae* was detected in the fluid of a borehole in South Africa and the SAGMA cluster was found in a study in rivers (Herfort et al., 2009; Hernández-Torres et al., 2015). The ecological significance and potential function of these two groups deserved to be further investigation in the future.

The phylum *Crenarchaeota*, accounted for 9.4–43.8% in TY soils, while only accounted for 1.03–14.1% in BH and LZ soils. The higher relative abundance of *Crenarchaeota* in sub-soil layers was mainly determined by the presence of the MCG group, which was previously found more abundant in subsurface soils with anoxic environments (Mikkonen et al., 2014), presumably due to their anaerobic characteristic. Similarly, some subgroups under this class were recently detected in river sediments and were reported to preferentially occupy deeper layers of the sediments with reducing conditions (Lazar et al., 2015).

The phylum *Euryarchaeota* was detected in all profile depths with the proportion varying between 9.7 and 44.6% and mainly composed of *Methanobacteria* and *Methanomicrobia* in this study. The proportions in the present study were clearly higher than the proportion (∼15%) in upland soils according to previously reported (Hu et al., 2013), as the representative *Euryarchaeota*-affiliating class *Methanobacteria* and *Methanomicrobia* were commonly detected in anaerobic environments such as aquatic ecosystems, wetlands, and paddy soils and responsible for methanogenesis (Mao et al., 2015; Tong et al., 2015). Although the two classes were both detected in all the samples, the ratio of *Methanobacteria* and *Methanomicrobia* proportion varied markedly among soils types, ranging from 22.22 in BH soils to 0.23 in TY soils. Similarly, it was observed that the relative abundance of *Methanomicrobia* increased largely in the anoxic zone in a lake while *Methanobacteria* was absent in a previous report (Hugoni et al., 2015). Conversely, *Methanobacteria* was widely identified in another study in a water-flooded oil reservoir without detecting *Methanomicrobia* (Hernández-Torres et al., 2015). All these imply that the two classes probably compete for niche and substrate and replace each other under specific soil conditions.

### Microbial Functional Structure in Different Paddy Soils and Linkage with Community Structure

Analyzing microbial functional genes of key enzymes related to biogeochemistry and metabolic pathways is essential to link microbial communities to their ecological functions. Functional gene array has been proved being a more effective approach to accomplish this task compared to conventional molecular ecology techniques (He et al., 2010, 2012; Kang et al., 2013). The detected genes in this study involved in diverse functions such as carbon degradation, methane oxidation and production, nitrogen and sulfur cycling, phosphorus utilization, heavy metal and antibiotic resistance, organic remediation and other categories, suggesting versatile ecological processes occurrence in these paddy soils. Revealing the pattern of these genes would be helpful to understand and predict relative functional processes performed by these gene categories (Bai et al., 2013; Zhang et al., 2013). However, DCA and SEMs analysis did not figure out similar effects from environmental factors on functional structure as the effect on bacterial community, except that the functional structure separated between the two rice growth stages in DCA plot (**Figure 1C**), suggesting the factors relative with plant growth potentially affected the structure of soil microbial functions to a greater degree even than soil type. Although significant effect of soil properties on microbial functional structure was not detected by SEMs, microbial functional gene diversity and abundance in the LZ soils (Oxisol) was significantly lower than in the BH soils (Inceptisol) and the TY soils (Ultisol) (**Figure 4** and **Table 3**), and the functional structure in the BH and TY soils were more similar compared to the LZ samples in DCA plot (**Figure 1**). Such pattern might be attributed to the complex influences from soil type but not to a single factor as suggested before (Cao et al., 2012; Zhao et al., 2016). On the other hand, significant effects of bacterial community structure on functional structure while the weak relationship between archaeal community and functional structure were detected by SEM (**Figure 6**), possibly due to the low amount of archaeal probes in the functional gene array analysis.

To establish the linkage for microbial community composition and their function, network analysis between microbial taxa and functional genes were generated in the present study, and the analysis has further proved the known abundant microbial groups such as α- and β*-proteobacteria* and *Acidobacteria* could play essential roles in regulating ecological functions. However, the networks also suggested that the predominated microbial groups in soils did not necessary provide intense functional potentials, while less abundant microbial groups might take greater parts in soil ecological processes. For instance, the two dominant Phyla, *Firmicute* and *Actinobacteria* were detected in all soils but their numbers of connections with functions only ranged between 2 and 11 in the networks. On the contrary, some groups with lower relative abundance such as ε-*Proteobacteria* and *Parvarchaota* had more than 32 and 157 connections with functions (Supplementary Tables 3, 4).

Although both bacterial and archaeal community composition shifted greatly along soil profiles, the general functional structure only varied among soil types but not across the profile depths in the present study. The possible reason for the distinctive pattern of microbial taxonomic and functional composition along soil profiles could be that some microbial groups with less abundant take greater parts in soil ecological processes as mentioned above. Furthermore, the result was consistent with previous observation that the change of microbial community composition does not necessarily connect to a change of microbial functions, and could be explained as the functional redundancy of soil microorganisms (Comte and Giorgio, 2010). Given the possibility that most of microbes inhabiting in soils possessed similar functional genes, the fluctuation in taxonomic structure along soil profile gradients would not necessarily alter the microbial function structure. Such ability could serve as a fundamental property of soil microbes which is essential to environmental perturbation (Comte and Giorgio, 2010). The similar weak linkages between microbial taxonomic and functional community structure were previously observed in Antarctic soils (Yergeau et al., 2007, 2012; Chong et al., 2015), subtropical broadleaf forests (Ding et al., 2015), fen peatlands (Haynes et al., 2015), and in microbial stream biofilms (Dopheide et al., 2015), which further corroborated the theory.

## Conclusion

By combining Illumina Mi-Seq sequencing with Geochip techniques, the study demonstrated manifest separation of bacterial and archaeal community and function structure among soil types, and visible but relatively slight shift of community structure along soil depth within each soil type, suggesting the overwhelming effect of soil parent material characteristics mainly via determination of soil pH, even under uniform rice cultivation management. Bacterial community showed significantly shifted along soil depth gradients mainly driven by organic carbon, while archaeal community variation along soil depth was mainly determined by salinity. Moreover, bacterial and archaeal taxa showed specific distribution patterns according to soil types or profiles, dependent on their ecophysiological properties. Especially, archaeal community composition showed contrasting patterns among the three paddy soils. Network analysis of bacterial and archaeal community indicated paddy soils harbored a higher degree of habitat heterogeneity for microbes in upper soils, and thus endowed microbes with higher system resistance and less intensive interaction in the surface soils compare to deeper soils. The relatively weak alignment between microbial community and functional structure suggested possible functional redundancy, and implied the potential resistance and resilience of microbial communities to environmental disturbance within these paddy soils.

## Author Contributions

RB was responsible to most of the laboratorial works, data processing, and article writing. J-TW conducted the construction of network analysis of microbial community and functional genes, and contributed to the analysis of sequencing data and construction of structure equation models. YD provided essential ideas to the article writing, as well as assistance in constructing phylogenetic molecular ecological networks. J-ZH provided essential ideas to the experimental design and article writing. KF contributed to the construction of phylogenetic molecular ecological networks. L-MZ provided essential ideas to the experimental design, and was responsible for mesocosm setup, article writing and revising.

## Conflict of Interest Statement

The authors declare that the research was conducted in the absence of any commercial or financial relationships that could be construed as a potential conflict of interest.

## Footnotes

1 http://ieg.ou.edu/microarray/

2 http://ieg.ou.edu
