# Supplementary material for: Microbial Community and Functional Structure Significantly Varied among Distinct Types of Paddy Soils But Responded Differently along Gradients of Soil Depth Layers
Source: Front Microbiol. 2017 May 29;8:945. doi: 10.3389/fmicb.2017.00945 (PMC5447084; doi:10.3389/fmicb.2017.00945)
Supplement: Supplementary file 1 [file Data_Sheet_1.DOCX]

**Supplementary Table** **1** **Pearson correlations between major bacterial groups and soil properties;** “*” denotes *P* < 0.05, “**”denotes *P* < 0.01

|  | *Acidobacteria* | *Actinbacteria* | *Bacteroidetes* | *Chloroflexi* | *Cyanobacteria* | *Firmicutes* | *Planctomycetes* | *Proteobacteria* | *Verrucomicrobia* |
| --- | --- | --- | --- | --- | --- | --- | --- | --- | --- |
| HWC | -0.15 | -0.458 | 0.06 | -0.62** | 0.2 | -0.56** | 0.29* | 0.46** | 0.51** |
| pH | -0.47** | 0.177 | 0.53** | 0.15 | -0.06 | -0.07 | -0.68** | 0.12 | -0.23 |
| NH_4_^+^ | -0.04 | 0.097 | -0.15 | -0.24 | -0.01 | -0.21 | 0 | 0.08 | 0.26* |
| NO_3_^+^ | -0.05 | 0.16 | 0 | -0.16 | 0.42** | 0.08 | 0.15 | -0.1 | 0.13 |
| EC | -0.47** | 0.525 | 0.32* | 0.27* | 0.21 | 0.15 | -0.62** | -0.11 | -0.263* |
| Depth | 0.42** | 0.051 | -0.29* | 0.42** | -0.55** | 0.43** | 0.15 | -0.33 | -0.377 |
| OM | -0.09 | -0.138 | -0.06 | -0.35** | 0.32* | -0.25* | 0.30* | 0.19 | 0.37** |
| S | -0.15 | 0.581 | -0.19 | 0.33* | 0.25* | 0.32* | -0.1 | -0.25 | -0.31* |
| AO-Fe | 0.39** | 0.275 | -0.53** | 0.39** | 0.07 | 0.45** | 0.27* | -0.41** | -0.18 |
| DCB-Fe | 0.09 | 0.588 | -0.42** | 0.54** | 0.17 | 0.55** | -0.03 | -0.43** | -0.45** |
| interMn | -0.19 | 0.578 | -0.1 | 0.57** | 0.11 | 0.42** | -0.40** | -0.28 | -0.53** |
| reducMn | -0.37** | 0.324 | 0.32* | 0.23 | -0.07 | 0.06 | -0.54** | -0.03 | -0.14 |
| Water | -0.42** | -0.092 | 0.13 | -0.49** | 0.59** | -0.36** | 0.16 | 0.34** | 0.39** |

**Supplementary Table** **2** **Pearson correlations between *Proteobacteria* classes and soil properties;** “*” denotes *P* < 0.05, “**”denotes *P* < 0.01

|  | α-*Proteobacteria* | β-*Proteobacteria* | δ-*Proteobacteria* | γ-*Proteobacteria* | ε-*Proteobacteria* | ζ-*Proteobacteria* |
| --- | --- | --- | --- | --- | --- | --- |
| HWC | .447** | .358* | 0.236 | 0.16 | -0.034 | -0.248 |
| pH | -.294* | .410** | 0.228 | .348* | .649** | 0.235 |
| NH4 | -0.069 | 0.225 | 0.12 | 0.116 | -0.096 | -0.073 |
| NO3 | 0.215 | 0.032 | -0.066 | 0.022 | -0.239 | -0.115 |
| EC | -.354* | 0.236 | -0.106 | 0.115 | .330* | .293* |
| Depth | -0.079 | -.511** | 0.075 | 0.076 | 0.144 | 0.221 |
| OM | 0.263 | 0.158 | -0.098 | -0.167 | -.318* | -0.096 |
| S | -0.264 | -0.104 | -.528** | -.418** | -.332* | 0.254 |
| AOFe | -0.234 | -.528** | -.582** | -.608** | -.596** | 0.026 |
| DCBFe | -0.338 | -.440** | -.757** | -.653** | -.511** | 0.176 |
| interMn | -.450** | -0.14 | -0.58 | -.401** | -0.106 | .298* |
| reducMn | -0.456 | 0.242 | 0.015 | 0.117 | .312* | 0.31 |
| Water | 0.082 | .542** | -0.172 | -0.123 | -0.191 | -0.22 |

**Supplementary Table 3 Number of edges between bacterial phylum and functions within each soil layers derived from mutualistic networks.** Bacterial groups mentioned in the text were marked in yellow color.

| Bacterial taxa | Layer A | Layer B | Layer C | Layer D |
| --- | --- | --- | --- | --- |
| Acidobacteria | 55 | 62 | 42 | 79 |
| Actinobacteria | 2 | 2 | 2 | 0 |
| α-Proteobacteria | 13 | 28 | 18 | 11 |
| Aquificae | 0 | 4 | 16 | 16 |
| Armatimonadetes | 2 | 7 | 2 | 1 |
| BRC1 | 13 | 4 | 11 | 3 |
| Bacteroidetes | 23 | 13 | 21 | 2 |
| β-Proteobacteria | 18 | 22 | 16 | 1 |
| Caldiserica | 3 | 6 | 0 | 2 |
| Chlamydiae | 10 | 36 | 12 | 18 |
| Chlorobi | 24 | 5 | 12 | 25 |
| Chloroflexi | 23 | 13 | 19 | 11 |
| Chrysiogenetes | 0 | 0 | 8 | 11 |
| Cyanobacteria | 1 | 5 | 3 | 0 |
| Deferribacteres | 2 | 7 | 42 | 53 |
| Deinococcus-Thermus | 6 | 7 | 10 | 2 |
| δ-Proteobacteria | 35 | 33 | 11 | 17 |
| Elusimicrobia | 8 | 3 | 15 | 14 |
| ε-Proteobacteria | 3 | 14 | 27 | 32 |
| Fibrobacteres | 3 | 0 | 0 | 0 |
| Firmicutes | 12 | 9 | 8 | 11 |
| Fusobacteria | 4 | 5 | 0 | 0 |
| γ-Proteobacteria | 55 | 1 | 4 | 1 |
| Gemmatimonadetes | 8 | 39 | 51 | 40 |
| Lentisphaerae | 8 | 14 | 4 | 2 |
| Nitrospira | 59 | 3 | 22 | 7 |
| OD1 | 6 | 32 | 4 | 3 |
| OP11 | 5 | 7 | 7 | 8 |
| Planctomycetes | 17 | 14 | 3 | 11 |
| SR1 | 59 | 0 | 11 | 0 |
| Spirochaetes | 12 | 1 | 11 | 11 |
| Synergistetes | 4 | 5 | 0 | 1 |
| TM7 | 0 | 0 | 0 | 0 |
| Tenericutes | 2 | 0 | 3 | 32 |
| Thermodesulfobacteria | 0 | 3 | 0 | 2 |
| Thermotogae | 2 | 6 | 1 | 0 |
| Verrucomicrobia | 19 | 4 | 8 | 15 |
| WS3 | 33 | 7 | 0 | 23 |

**Supplementary Table 4 Number of edges between archaeal taxa and functions within each soil layers derived from mutualistic networks**. Archaeal group mentioned in the text were marked in red color.

| Archaeal taxa | Layer A | Layer B | Layer C | Layer D |
| --- | --- | --- | --- | --- |
| Eurtarchaeota/Methnobacteria | 27 | 17 | 6 | 46 |
| Eurtarchaeota/Methnomicrobia | 67 | 110 | 66 | 86 |
| Eurtarchaeota/Thermoplasmata | 72 | 42 | 70 | 65 |
| Thaumarchaeota/Cenarchaeaceae | 0 | 11 | 52 | 6 |
| Thaumarchaeota/SAGMX | 4 | 10 | 17 | 13 |
| Thaumarchaeota/Nitro | 16 | 32 | 15 | 13 |
| Crenarchaeota/MBGA | 19 | 78 | 11 | 19 |
| Crenarchaeota/MCG | 50 | 23 | 14 | 113 |
| Parvarchaeota | 20 | 157 | 40 | 56 |


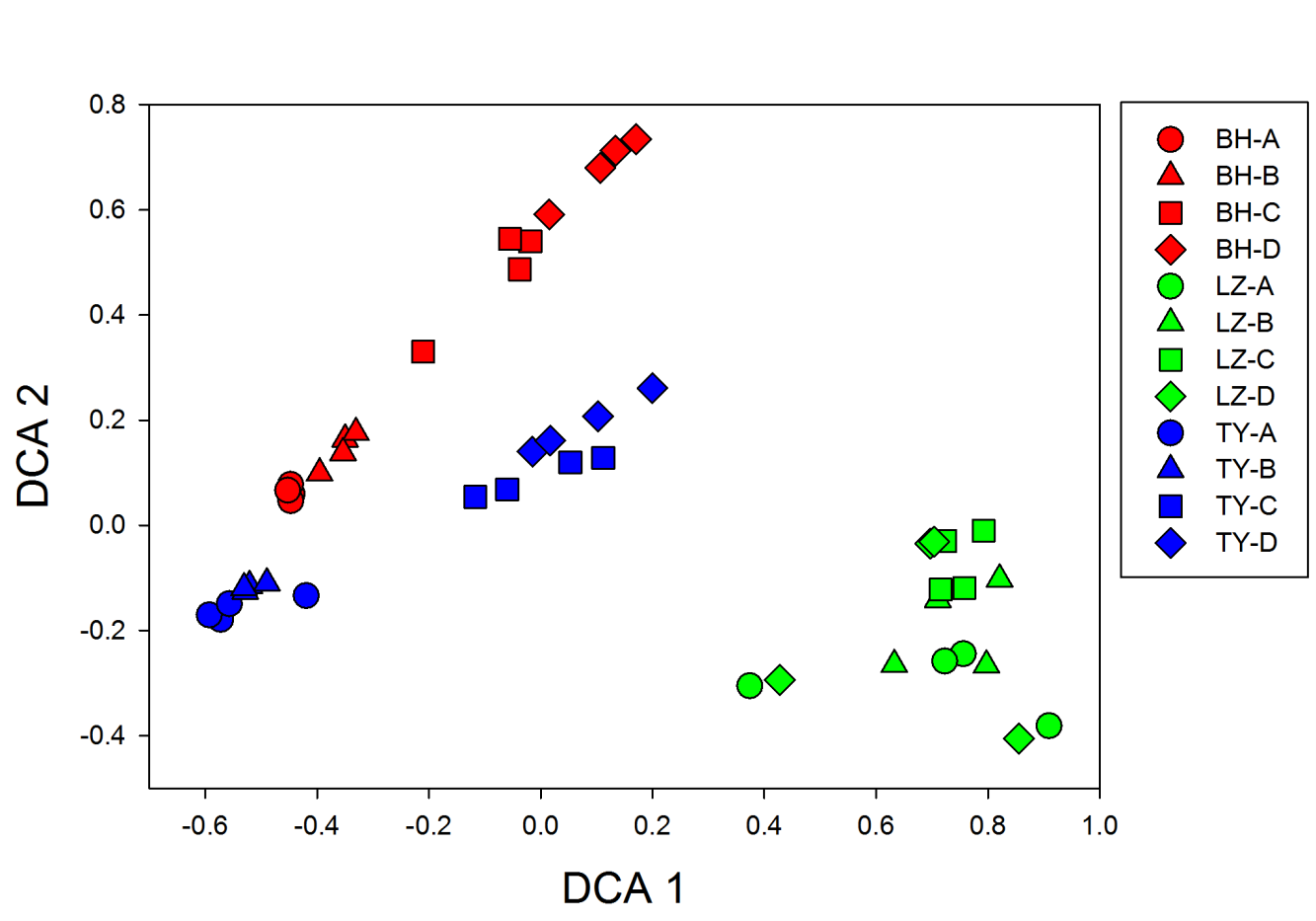


**Supplementary Figure 1. DCA of soil properties in the three paddy soils**


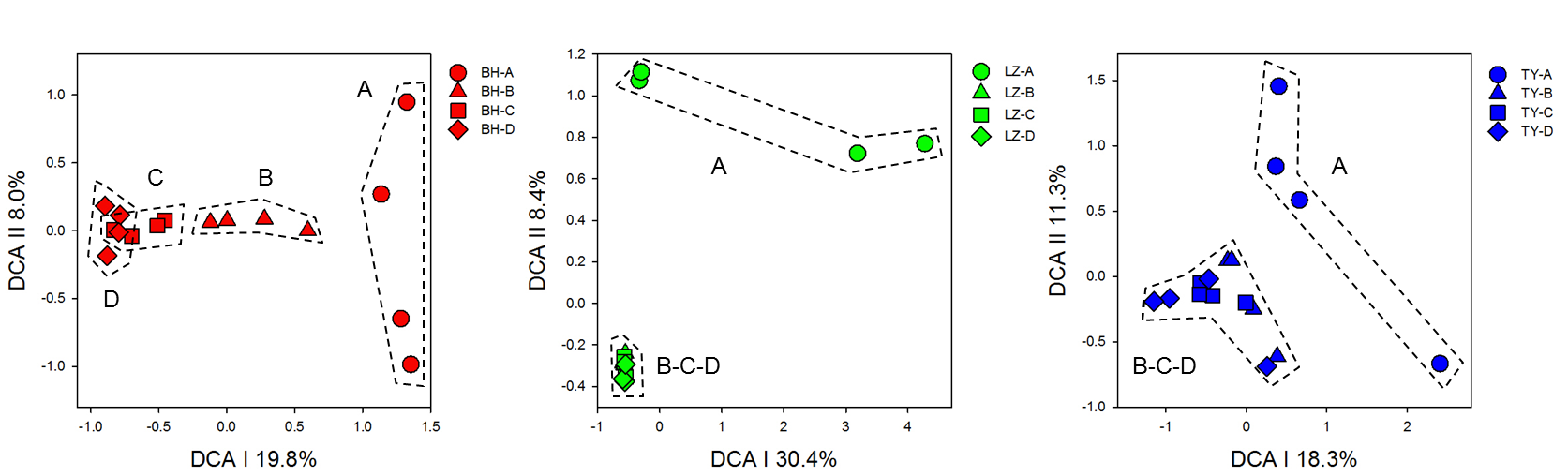


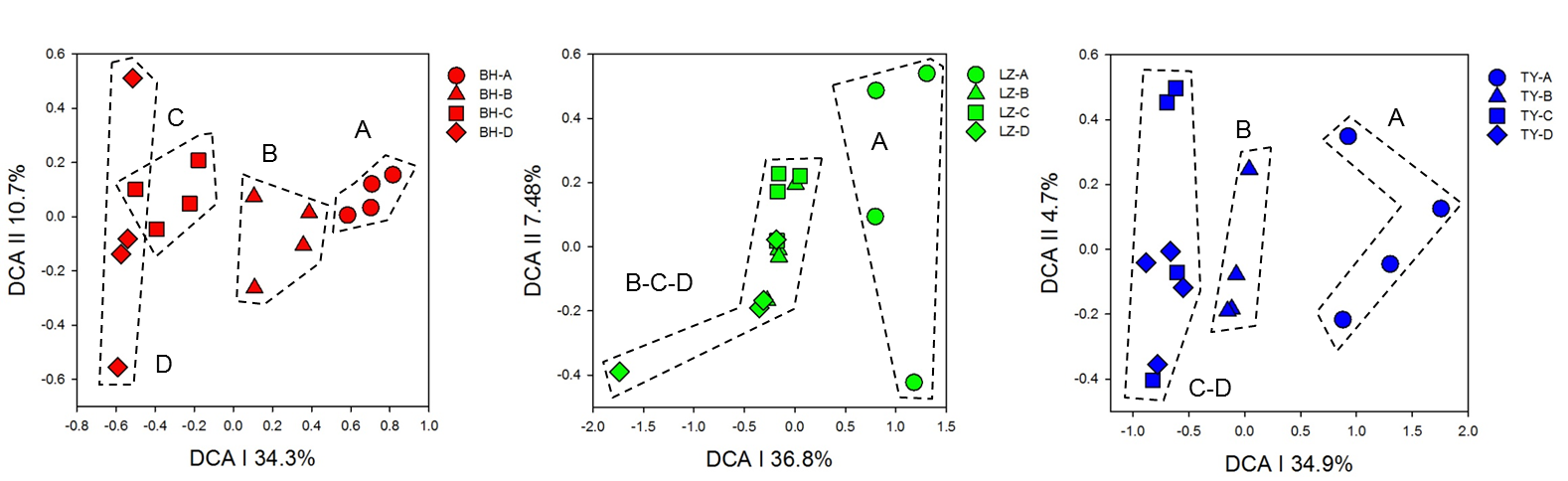


**
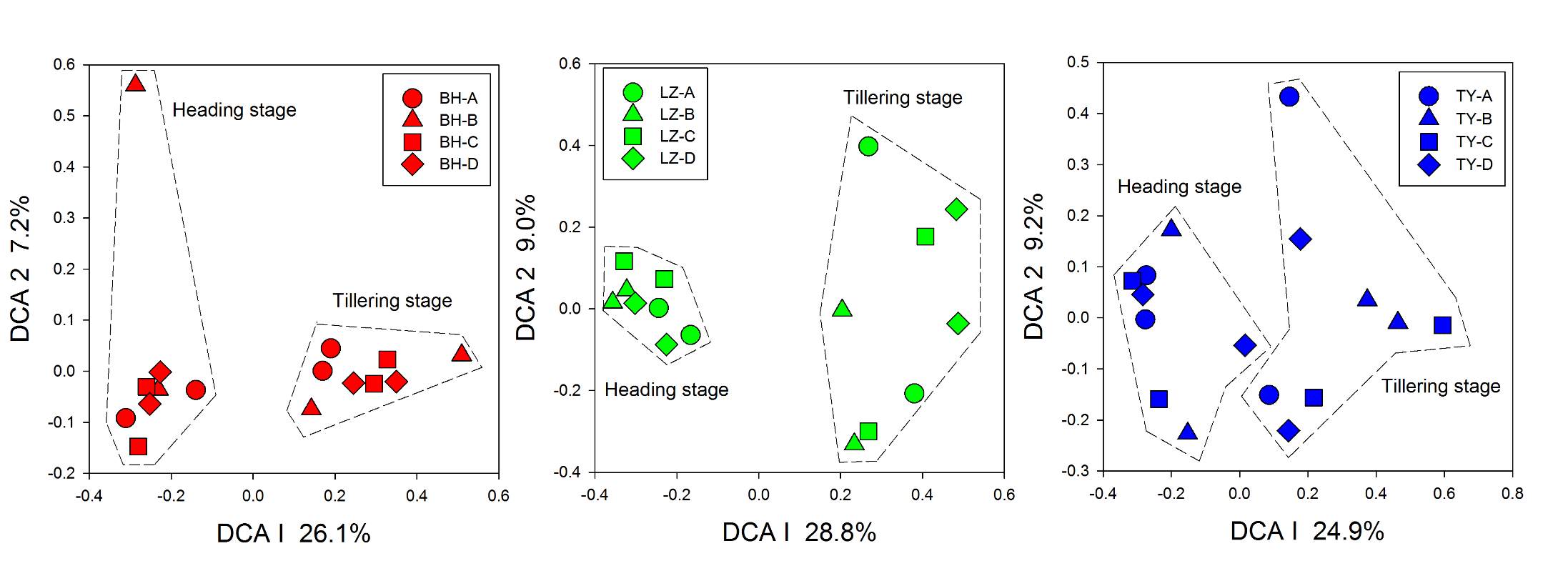
**

**Supplementary Figure 2. DCA for community structure of bacterial (top), archaeal (middle) community structure (based on the 97% level of OTU) and functional genes (bottom) in each paddy soil.**

(A)

(B)

**Supplementary Figure 3. Standardized total effects of factors on bacterial (A) and archaeal (B) community derived from standardized structural equation model.**
